# A case report of aggressive course of CD30+ primary cutaneous anaplastic large cell lymphoma

**DOI:** 10.1097/MD.0000000000025770

**Published:** 2021-05-07

**Authors:** Wen-Tian Lyu, Qi-Bin Song, Wang Qiong, Jing Liu, Ren Yong, Feng-Tao Yi, Dong-liang Han

**Affiliations:** a Department of Oncology, Huanggang Center Hospital, Huangzhou District, Huanggang; b Department of Pathology; c Department of Radiation Oncology, General Hospital of Central Treater Command of PLA, Hongshan District, Wuhan, China.

**Keywords:** aggressive behavior, CD30+ primary cutaneous anaplastic large cell lymphoma, clinical manifestation, pathology, radiotherapy

## Abstract

**Introduction:**

CD30+ primary cutaneous anaplastic large cell lymphoma (PC-ALCL) is a rare T-cell neoplasm, and has been reported to present with an indolent behavior. The PC-ALCL with aggressive behavior has not been reported in the literature.

**Patient concerns:**

We treated a patient with PC-ALCL that exhibited indolent behavior in the past 2 years and aggressive behavior within the last 3 months before presentation.

**Diagnosis:**

Aggressive CD30+ primary cutaneous anaplastic large cell lymphoma.

**Interventions:**

The radiotherapy regimen was individualized in terms of the target volume delineation and dose prescription, and the dose–response relationship was evaluated.

**Outcomes:**

The mean distance of microscopic infiltration was 14.1 mm in depth and 14.3 mm circumferentially. The lesion completely regressed after the delivery of 40 Gy in 20 fractions over 4 weeks. The tumor did not recur over the next year.

**Conclusion:**

An aggressive disease course is rare for indolent CD30+ PC-ALCL, which has similar histopathological characteristics as indolent PC-ALCL. The radiotherapy strategy should be individualized with curative intent.

## 1 Introduction

CD30+ primary cutaneous anaplastic large cell lymphoma (PC-ALCL) is a rare ALK-negative T-cell neoplasm with an annual incidence of 10 per million persons.^[1]^ Patients with this malignancy usually present with indolent solitary or localized nodules with or without ulcerative lesions. PC-ALCL with indolent behavior is radiosensitive, as indicated by its 95% complete clinical response rate and 90% 5-year disease-specific survival rate.^[2,3]^ Most studies about radiotherapy for PC-ALCL were retrospective analyses, and these studies examined PC-ALCL with indolent behavior and assessed the relationship between clinical response and the radiation dose. Involved-site radiotherapy has been recommended as the appropriate modality for treating primary cutaneous lymphoma.^[4–7]^ It is necessary to consider individualized radiotherapy regimens for rare tumor types. We recently received a patient who developed an ulcerative lesion on the neck skin 3 months before presentation, and this lesion arose from an indolent node over the preceding 2 years. The pathological result was consistent with normal PC-ALCL, which usually exhibits indolent behavior. However, both the lesion and the magnetic resonance imaging (MRI) revealed an aggressive nature that had not been described in the literature. The radiotherapy strategy was individualized with curative intent.

### 1.1 Patient information/clinical findings

**Figure 1:**
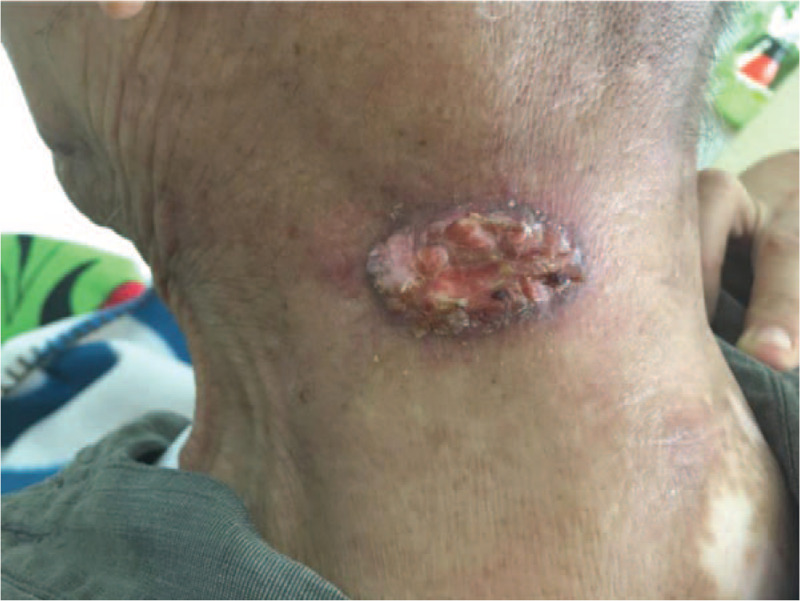
A progressive ulcerative lesion that was treated with radiotherapy at a dose of 8 Gy delivered in four fractions.

A 91-year-old paralyzed male with a 20-year history of hypertension presented with a rapidly growing ulcerated lesion on the skin of his neck (40 × 30 mm^2^). Figure 1 presents the lesion, which was treated with radiotherapy (8 Gy in 4 fractions). This lesion began as an intact nodule that had appeared >2 years before presentation, and it began to ulcerate in the last 3 months before presentation. The rupture, which was nonpainful, had rapidly grown. Computed tomography (CT) revealed no extranodular lesions. The Karnofsky score was 40. The patient was not admitted to the hospital, and he returned home after each radiotherapy session.

### 1.2 Diagnostic assessment and therapeutic interventions

**Figure 2:**
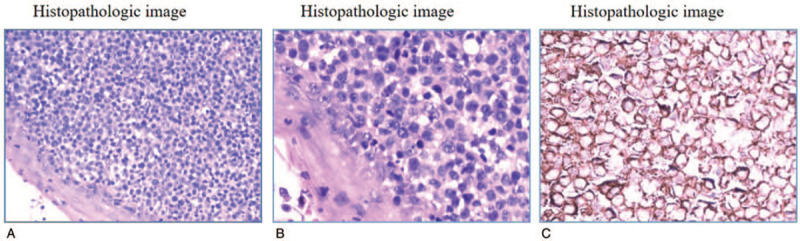
Hematoxylin-eosin staining. (A) The dermis was densely infiltrated by atypical lymphocytes without involvement of the epidermis (original magnification, ×200). B. The dermis was densely infiltrated by atypical lymphocytes, which mostly consisted of anaplastic large cells (original magnification, ×400). (C) CD30 staining was completely positive (original magnification, ×400).

This study was approved by the ethic committee of Huanggang Center Hospital. With the informed consent of the patient's family, we conducted the treatment and study on this patient. Core needle biopsy of the lesion was performed to examine the morphology of the neoplastic cells via hematoxylin-eosin (HE) staining. The expression of CD45, CD4, CD30, CD19, CD20, Pax-5, CD3, CD5, CD8, EMA, perforin, CD56, CD34, and ALK in the neoplastic cells was analyzed. According to HE staining, the microscopic appearance of this tumor indicated the presence of anaplastic large cells in the dermis and subcutaneous tissue. Immunohistochemistry revealed that the lesion was negative for CD20, CD8, CD3, ALK, and S-100 and positive for CD4 and Ki-67 (50%). CD30 positivity was identified in all cells. The pathological findings were consistent with a diagnosis of PC-ALCL (Fig. 2).

**Figure 3:**
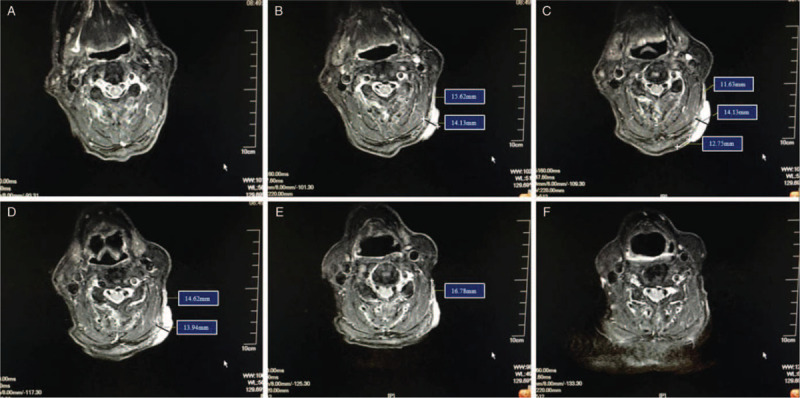
Magnetic resonance imaging of aggressive CD30+ primary cutaneous anaplastic large cell lymphoma. The T2 sequence revealed a lesion that protruded (white arrow) from the skin. The thin areas (yellow arrow) represented the subcutaneous part of the tumor. The images in A–F revealed the extent of invasion by depth and circumference.

A 1.5 Tesla MR system was used to scan the neck. The scanning section thickness was 3 mm. T1- and T2-weighted images were used to define the gross tumor volume and CTV. T2-weighted MRI demonstrated that the invisible part of this lesion resided subcutaneously, and its extent of invasion differed among the three dimensions. The visible part of the lesion measured 14.1 mm in depth on average. The invisible part that developed from microscopic infiltration and resided subcutaneously measured 14.3 mm circumferentially (Fig. 3).

The clinical manifestation of this tumor was extremely different from the typical manifestation of PC-ALCL, which usually has indolent characteristics. The ulcerative lesion in this case was characterized as a continuous expansion of skin lesions that began as an indolent nodule appearing 2 years before presentation and progressing to an ulcerative lesion of 40 × 30 mm^2^ during the previous 3 months. The histopathology of the lesion was completely consistent with that of CD30+ PC-ALCL. And MRI also revealed that the tumor was aggressive with subcutaneous infiltration up to 14 mm. In summary, the tumor was clinically diagnosed as aggressive CD30+ PC-ALCL, which originated from an indolent CD30+ PC-ALCL. According to the ISCL/EORTC TNM classification, the tumor was staged as T1aN0M0.^[8]^

Due to the early stage and high radiosensitivity of this lymphoma, a radiotherapy regimen with a radical intent was figured out. Radiation was delivered using a Varian 600 C/D linear accelerator. The Pinnacle treatment planning system was used in this work. The head and neck of the patient were immobilized through CT simulation. A 1-cm-thick bolus was placed between the headrest and the site at which the tumor was located. CT images (3-mm-thick sections) were sent to the treatment planning system. T2-weighted sequences plus a 2-mm margin defined the bulk of the microscopic infiltration (clinical target volume, CTV). The CTV plus a 3-mm setup error was used to determine the planning target volume (PTV). Involved-site radiotherapy was achieved using this scheme. And a 6-Mev electron ray was selected to treat the lymphoma.

**Figure 4:**
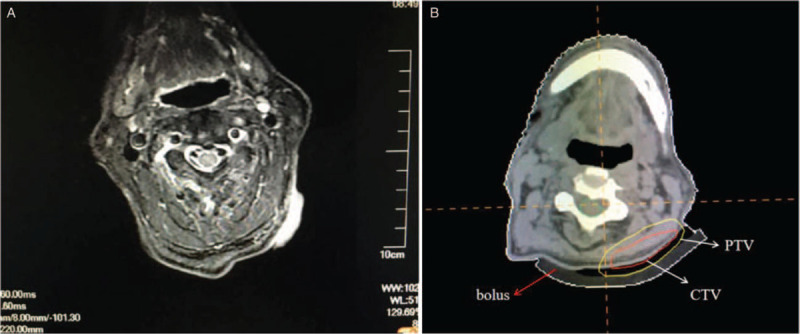
Radiotherapy target volume delineation. (A) T2-weighted magnetic resonance imaging (MRI) of the neck was used to delineate the target volume of the tumor. (B) With reference to MRI of the same anatomical section, the target volume of radiotherapy was contoured onto the computed tomography image of the treatment planning system. The red arrow-marked bolus was used to enhance the dose delivered to the skin. The red circle referred to the clinical target volume (CTV). The yellow circle referred to the planning target volume (PTV).

**Figure 5:**
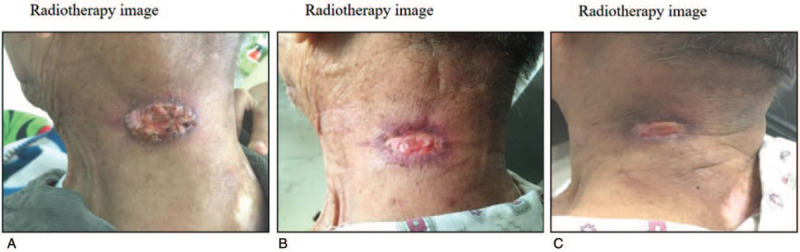
The presentation of aggressive CD30+ primary cutaneous anaplastic large cell lymphoma and the treatment response. The tumor was treated via radiotherapy using the following protocols: 8 Gy in four fractions (A), 20 Gy in 10 fractions (B), and 34 Gy in 17 fractions (C).

The radiation dose was 40 Gy delivered in 2-Gy fractions over 4 weeks (5 fractions/week). The radiation was targeted to the PTV (Fig. 4). The lesion was nearly eradicated by the radiotherapy regimen (40 Gy in 20 fractions, Fig. 5). One week after the completion of treatment, the ulceration had healed completely. The tumor did not relapse during 1 year of follow-up before the patient's death due to cerebral infarction.

## 2 Discussion

Primary cutaneous T-cell lymphoma is a type of primary cutaneous lymphoma, which includes primary cutaneous CD30+ T-cell lymphoproliferative disorders (PCTLDs), mycosis fungoides (MF), and Sézary syndrome.^[7,8]^ PCTLDs have customarily been classified on the basis of their clinical presentation as lymphomatoid papulosis, PC-ALCL, and borderline cases with overlapping clinical and histopathological features.^[9]^

PC-ALCL is a rare, generally indolent cancer with a favorable prognosis including a 5-year disease-specific survival rate of 90%.^[2,3]^ Spontaneous or partial tumor remission occurs in 44% of cases. On histopathology, the lesions feature large anaplastic, pleomorphic, or immunoblastic T-cells that express CD30 (a cell membrane protein of the tumor necrosis factor receptor family). These cells display a large, irregular polygonal shape, and they are arranged in sheets extending throughout the dermis and sometimes the subcutis. Additionally, epidermotropism and adnexal involvement can be observed.^[10]^ Tumor involvement may be restricted to cutaneous lymphatics, and dermal lymphatic involvement is a frequent finding.^[11–14]^ CD30 expression in a membranous and Golgi pattern in >75% of tumor cells is definitional.^[15,16]^

Establishing a diagnosis of PC-ALCL requires a clinicopathologic correlation to distinguish this entity from other PCTLDs. PC-ALCL has histopathological and immunophenotypic similarities with systemic ALCL, but it arises primarily in the skin and features a more indolent behavior. The clinical behavior of lymphomatoid papulosis, characterized by recurrent and regressing crops of papules and nodules, distinguishes this malignancy from PC-ALCL.^[17]^ PC-ALCL and MF can have the same clinical presentation. The differential diagnosis between PC-ALCL and CD30+ MF depends on histopathological differences. PC-ALCL can also arise from the progression of MF.^[18]^

In this study, the ulcerative lesion arose from an intact node on the neck that initially developed 2 years before presentation and began to rapidly rupture within the last 3 months before presentation. Other than the discomfort caused by ulceration and infection, the patient did not feel any pain. And the MRI revealed that the mean distance of microscopic infiltration was 14.3 mm. These clinical characteristics confirmed the aggressive behavior of this lesion, which might have been the result of indolent PC-ALCL progression.

The histopathological studies in this work demonstrated that this aggressive lymphoma consisted of typical anaplastic cells morphologically, and its immunohistochemical findings were completely consistent with normal PC-ALCL, which usually has an indolent manifestation. A transformation from indolent behavior to aggressive behavior has not been reported in the literature. The histopathological difference between aggressive and indolent PC-ALCL is unclear. Exploration of special phenotypes and perforin expression may be helpful for distinguishing these malignancies.

PC-ALCL is a radiation-sensitive tumor with a clinical complete response rate of 95%. Because of its rarity, most knowledge about radiotherapy for PC-ALCL was derived from retrospective studies that focused on indolent PC-ALCL and examined the relationship between clinical response and the radiation dose. Therefore, defining the treatment volume of aggressive PC-ALCL targeted by radiation was a key point of this study. The radiation field is recommended to encompass the original suspicious lesions plus a 1- to 2-cm margin for curative treatment.^[4–7]^ In clinical practice, the microscopic infiltration distance of the tumor should be considered individually depending on the biological characteristics of the lesion. In this study, T2-weighted MRI, which assessed tumor-induced inflammatory edema, was used to demonstrate the extent of microinvasion. The T2 sequence margin plus an additional 2-mm margin was applied to define the CTV. Additionally, the CTV plus a 3-mm setup error comprised the PTV, which represented the target of radiotherapy.

In addition to defining the target volume, the dose prescription is another key point in radiotherapy. Yale Center records from 2008 indicated that radiotherapy was exclusively delivered at a dose of 34 to 44 Gy in 2-Gy fractions.^[19]^ The International Lymphoma Radiation Oncology Group analyzed 56 patients recruited from eight collaborating institutions and reported a recommended dose of 30 Gy for good local control.^[18]^ The National Comprehensive Cancer Network Clinical Practice Guideline in Oncology (version 1. 2021) recommended a total dose of 24 to 36 Gy for PC-ALCL.^[20]^ All dose regimens were developed for indolent PC-ALCL. However, these regimens must be individualized for patients, particularly those with aggressive PC-ALCL, which lacks an evidence-based radiotherapy strategy. We assessed the clinical response to radiotherapy daily in this work and found that the lesion quickly responded to the treatment. We observed that the lesion had regressed almost completely after the delivery of 40 Gy of radiation in 20 fractions. Four days after radiotherapy, the lesion was undetectable. No recurrence occurred during 1 year of follow-up.

One limitation of this work was that the 91-year-old paralyzed patient, who had a history of cerebral infarction, and was not admitted to the hospital during radiotherapy. It was difficult to visit the patient during ward rounds to obtain sufficient numbers of images, and it was not convenient for the patient to return to the hospital for follow-up examination. All follow-up consultations were conducted by telephone. Thus, we could not obtain sufficient pictures of the lesion to present in this article including the pictures taken before radiotherapy and the pictures after radiotherapy. In addition, the patient died of cerebral infarction 1 year after radiotherapy, and therefore, progression-free and overall survival could not be evaluated.

## 3 Conclusions

Aggressive CD30+ PC-ALCL can arise from indolent PC-ALCL. The mechanism of this transformation is unclear. The diagnosis should be based on the combination of pathology, clinical behavior and MRI. Individualized radiotherapy can be applied with curative intent.

## Author contributions

**Conceptualization:** Wen-Tian Lyu.

**Data curation:** Wen-Tian Lyu, Wang Qiong, Ren Yong, Dong-liang Han.

**Formal analysis:** Wen-Tian Lyu, Wang Qiong, Ren Yong.

**Methodology:** Wen-Tian Lyu, Qi-Bin Song, Ren Yong.

**Project administration:** Wen-Tian Lyu, Qi-Bin Song, Jing Liu.

**Resources:** Feng-Tao Yi, Dong-liang Han.

**Supervision:** Wen-Tian Lyu.

**Visualization:** Wen-Tian Lyu.

**Writing – original draft:** Wen-Tian Lyu.

**Writing – review & editing:** Wen-Tian Lyu.
